# Ultrasonication time dependent structuring of heat-treated legume proteins: interfacial adsorption and stabilization of faba bean and pea protein isolates in high internal phase Pickering emulsions

**DOI:** 10.1016/j.ultsonch.2025.107659

**Published:** 2025-10-30

**Authors:** Hyo Gyeong Lee, Jiseon Lee, Yeon-Ji Jo, Mi-Jung Choi

**Affiliations:** a Department of Food Science and Biotechnology of Animal Resources, Konkuk University, Seoul 05029, Republic of Korea; b School of Animal & Food Sciences and Marketing, Konkuk University, Seoul 05029, Republic of Korea; c Department of Marine Bio Food Science, Gangneung-Wonju National University, Gangneung 25457 Gangwon, Republic of Korea

**Keywords:** Faba bean protein isolate, Pea protein isolate, High internal phase, Pickering emulsions, Heat treatment, Ultrasonication treatment, Interfacial adsorption

## Abstract

This study investigated ultrasonication time dependent restructuring of heat-treated legume proteins, focusing on how sequential heat and ultrasound (HU) treatments influence the interfacial adsorption and stabilization of faba bean protein isolates (FBPIs) and pea protein isolates (PPIs) in high internal phase Pickering emulsions (HIPPEs). Proteins were first modified by heat treatment (90 °C, 2 h) and subsequently subjected to ultrasonication (20 kHz, 120 W, 5–20 min). The resulting samples were characterized in terms of particle size, solubility, turbidity, surface charge, and secondary structure. HU treatment significantly reduced protein aggregation and particle size from 16.44 μm (FCON) to 191.20 nm (FHU20) for FBPI, and from 48.25 μm (PCON) to 279.27 nm (PHU20) for PPI, while solubility increased from 59.29 % to 89.42 % in FBPIs and 38.16 % to 79.85 % in PPIs. Moderate sonication durations (5–10 min) yielded the smallest particles, highest solubility, and most flexible secondary structures, enabling cohesive and elastic interfacial films. By contrast, prolonged sonication (20 min) led to partial reaggregation, weakening interfacial efficiency. HIPPEs stabilized by untreated proteins exhibited larger, heterogeneous droplets, whereas those stabilized by HU-treated proteins exhibited smaller, uniform droplet distributions. Despite the reduced adsorbed protein at interface (AP) and interfacial protein concentration (*Γ*), CLSM and TEM images confirmed continuous and uniform interfacial layers by the HU-treated proteins, contributing to improved emulsion stability. Rheological analysis further demonstrated the enhanced gel-like structure and viscoelasticity. Overall, the study highlights ultrasonication duration as a critical variable in HU treatment, dictating colloidal restructuring and interfacial adsorption behavior, and underscoring the suitability of legume proteins as clean-label stabilizers for high-oil emulsion systems.

## Graphical abstract

**Figure:**
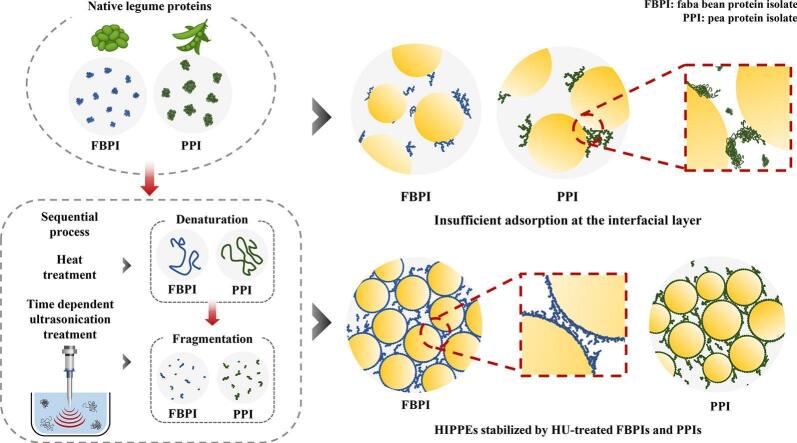

## 1 Introduction

Pickering emulsions (PEs) are emulsified systems stabilized by solid particles that are irreversibly adsorbed at the oil–water interface, forming a dense interfacial barrier that prevents droplet coalescence and enhances emulsion stability [1]. Unlike conventional emulsifiers, which are typically small-molecule surfactants, Pickering particles stabilize emulsions through steric hindrance and capillary interactions, rather than by reducing the interfacial tension [2]. Compared to conventional emulsions, emulsions stabilized by colloidal nanoparticles demonstrate superior resistance to coalescence and Ostwald ripening, improved oxidative stability, and enhanced encapsulation and controlled release of bioactive compounds [3,4]. These features make PEs particularly attractive for application in food, cosmetics, and pharmaceuticals, especially as the demand for clean labels and stable delivery systems continues to grow [5].

An ideal Pickering stabilizer must be partially wetted by both aqueous and oil phases, with particle sizes significantly smaller than the emulsion droplets, typically in the nano- to low-micrometer range, to ensure sufficient interfacial packing [6]. Protein particles offer several advantages. They are amphiphilic, biodegradable, biocompatible, and naturally abundant [7]. Their adjustable conformation and interfacial activity allow effective droplet coverage, particularly when proteins are transformed into colloidal aggregates or nanoparticles with appropriate wettability. Native proteins tend to unfold and aggregate at the oil–water interface, which can weaken emulsion stability [2]. In contrast, protein nanoparticles and microgels produced by physical or chemical crosslinking at high protein concentrations maintain their structural integrity during interfacial adsorption. This structural stability, combined with low toxicity and renewability, makes them promising candidates for use in food-grade PEs [8].

However, the stabilization capacity of protein nanoparticles depends critically on their surface wettability. Overly hydrophobic particles may fail to efficiently adsorb at the interface, whereas excessively hydrophilic particles may lack adequate interfacial anchoring [9]. To address this, various modification methods, such as pH-shifting [10], formation of hybrid protein-based particles through protein-polysaccharide [11,12], and protein-protein [13] interactions, and phenolic complexation [14,15] have been applied to optimize interfacial activity. These approaches have been shown to improve PE stability and functionality, often outperforming single-component protein systems, by enhancing interfacial adsorption and structural integrity.

Plant proteins are promising candidates for PE stabilization because of their sustainable sourcing, low cost, and functional diversity. Legume-derived proteins such as faba bean and pea proteins are rich in storage globulins, which are responsible for most functional attributes, including solubility and emulsifying activity [16]. Faba bean protein isolate (FBPI) is derived from the seeds of *Vicia faba*, a leguminous crop valued for its high protein content (approximately 29 %), excellent nitrogen-fixation capacity, and adaptability to diverse climates, making it a sustainable option for plant-based protein sourcing [17]. Nutritionally, faba bean protein is rich in essential amino acids and has potential for human and animal nutrition. Pea protein isolate (PPI), extracted from *Pisum sativum*, has garnered widespread use in food product development because of its high nutritional quality, low allergenicity, non-GMO status, and economic viability [16,18]. The increasing demand for PPI has paralleled the expansion of plant-based food markets. Both FBPI and PPI are globulin-rich isolates primarily composed of vicilin (7S) and legumin (11S). While vicilin offers flexibility and solubility, the compact structure and disulfide bonds of legumin reduce interfacial activity, highlighting the need for structural modification [19,20].

Despite these favorable compositional traits, the intrinsic globular compactness and limited conformational flexibility of native FBPI and PPI restrict their interfacial performance as effective PE stabilizers. In particular, the dense globular conformation, high molecular weight, and disulfide bonding characteristics of legumin-rich fractions can restrict solubility, reduce molecular flexibility, and hinder adsorption at the oil–water interface [21]. Such structural rigidity coupled with limited surface activity often leads to insufficient interfacial coverage and reduced emulsion stability in practical applications. To overcome these challenges, physical modification has emerged as an effective strategy for tailoring the structural and interfacial properties of plant proteins without chemical additives [10,22].

Among these strategies, physical modification techniques such as heat treatment and ultrasonication have shown promise. Heat treatment induces partial unfolding, exposing buried hydrophobic and sulfhydryl groups, and can lead to the disaggregation of protein subunits depending on the process parameters [23]. Ultrasonication applies high-frequency mechanical energy to generate localized cavitation, which disrupts non-covalent interactions and reduces the protein particle size [24]. Consequently, both techniques enhance solubility, surface activity, and protein dispersion, which are beneficial for interfacial adsorption and emulsion formation.

Proteins were selected in this study as potential Pickering stabilizers to align with the growing demand for clean-label formulations that avoid synthetic emulsifiers [5]. Their inherent amphiphilic nature enables adsorption at the oil–water interface, where they can unfold and rearrange to form stabilizing films, while also providing biodegradability and nutritional value. Plant proteins, in particular, are highly sustainable and compatible with consumer preferences, making them attractive alternatives for food-grade emulsion systems.

Given that the effectiveness of physical treatments such as heat and ultrasonication strongly depends on the intrinsic structural features of the protein source, it is important to select models that share similar storage globulin composition but differ in their responsiveness to modification. FBPI and PPI were therefore selected as representative legume proteins, both primarily composed of 7S vicilin and 11S legumin globulins [19,20], yet differing in structural flexibility and aggregation tendency, factors that critically influence their response to ultrasound. Previous studies have demonstrated that high-intensity ultrasound markedly reduces particle size, increases solubility, and enhances interfacial adsorption in FBPI by disrupting noncovalent aggregates and promoting partial unfolding [75,76]. Similarly, PPI exhibits strong ultrasound responsiveness, showing improved solubility and emulsifying behavior due to enhanced molecular mobility and surface reorganization [37,77].

In contrast, soy proteins, particularly the 11S glycinin fraction, display limited structural rearrangement under comparable ultrasonication conditions, leading to only modest improvements in interfacial performance [50]. Lentil proteins, while also responsive to ultrasound, have mainly been investigated in dilute dispersions or extraction contexts rather than under high internal phase conditions, leaving their interfacial behavior in densely packed systems largely unexplored [78]. These distinctions make FBPI and PPI suitable model systems for examining how sequential HU treatment alters molecular organization and interfacial film formation in emulsion system. In addition, both proteins are widely available, non-GMO, and already used in commercial food formulations [16], ensuring that the findings possess both mechanistic depth and practical relevance for clean-label emulsion design.

Combined heat and ultrasound (HU) treatment synergistically improves plant protein properties, including solubility, hydrophobicity, surface charge, and emulsifying capacity [10]. Although numerous studies have investigated the use of either heat or ultrasound individually to modify legume and other plant proteins, and a few have explored their combined application [10,44,45]. These investigations have primarily focused on improving solubility, digestibility, or surface activity in dilute aqueous dispersions or conventional O/W emulsions. However, the behavior of proteins subjected to combined heat-ultrasound (HU) treatment under the highly constrained conditions of high internal phase Pickering emulsions (HIPPEs, φ > 0.7) remains largely unexplored. In HIPPEs, the stabilizing particles must form continuous, cohesive, and elastic interfacial films to resist droplet deformation and coalescence, requirements far more demanding than those in conventional emulsions.

In this study, HU treatment was employed as a structural control strategy to elucidate how ultrasonication duration modulates the restructuring of legume proteins and governs interfacial film formation in HIPPEs. Particular attention was paid to the non-linear evolution of solubility, particle size, and secondary structure, especially the unfolding, reaggregation transition beyond 10 min, and how these structural transitions determine interfacial continuity and rheological strength. Importantly, our findings demonstrate that film cohesion and elasticity, rather than the total amount of adsorbed protein, are the dominant determinants of emulsion stability, challenging the conventional assumption that greater interfacial coverage directly translates into enhanced stability. Furthermore, by comparing two structurally similar but functionally distinct legume proteins, FBPI and PPI, we provide mechanistic insights into how molecular flexibility and disulfide bond rearrangement regulate film formation efficiency in HU-treated HIPPE systems.

## 2 Materials and methods

### 2.1 Materials

FBPI was obtained from Cosun Protein (Dinteloord, Netherlands), and PPI was purchased from Kwangil Co., Ltd. (Seoul, Korea). Canola oil was purchased from Sajohaepyo Corporation (Seoul, Korea). All the chemical products were of analytical grade.

### 2.2 Physical modification of legume protein isolates

FBPI and PPI dispersions were prepared by dispersing each protein isolate (4 % w/v) in distilled water containing sodium azide (0.02 %, w/v) to prevent microbial growth. The suspensions were stirred for 2 h at ambient temperature and allowed to equilibrate overnight at 4 °C. The pH was maintained at approximately 7, which was above the isoelectric point (pI) of both proteins, to promote a negative surface charge and minimize spontaneous aggregation.

Following equilibration, the dispersions were subjected to two-step physical modification. First, a heat treatment was performed in a water bath at 90°C for 2 h. After heating, the samples were rapidly cooled in an ice bath to stabilize denatured proteins. Subsequently, protein dispersions (100 mL) were subjected to ultrasonication using an ultrasonic homogenizer (HF-GM 2200, BANDELIN Electronics GmbH & Co. KG, Berlin, Germany) equipped with a titanium microtip (MS-73, 3 mm diameter). The probe was immersed approximately 20 mm below the liquid surface and positioned centrally in a 250 mL glass beaker to minimize surface cavitation and wall effects. Sonication was applied at a nominal power of 120 W and frequency of 20 kHz for 5, 10, or 20 min in pulse mode (9 s on, 1 s off). To suppress heat accumulation, the beaker was maintained in an ice water bath, and the sample temperature was monitored before and after treatment to ensure it remained below 10°C. Samples were designated according to protein type and treatment: FCON and PCON (untreated controls), and FHU5, FHU10, FHU20 or PHU5, PHU10, PHU20 (heat-treated followed by 5, 10, or 20 min of ultrasonication for FBPI and PPI, respectively).

The selection of temperature and ultrasonic power was based on optimization results from our previous study on legume proteins [23], where a heating temperature of 90 °C for 2 h and an ultrasonic power of 120 W (20 kHz) were identified as effective conditions for partially unfolding storage globulins while minimizing irreversible aggregation or degradation. This combination provided sufficient cavitation intensity to disaggregate protein particles and expose hydrophobic residues, while preserving structural integrity and solubility. Therefore, the same parameters were adopted in this study to ensure comparability with prior findings and to systematically evaluate the effect of ultrasonication time on protein restructuring and interfacial functionality in HIPPEs.

### 2.3 Properties of HU-treated legume protein isolates

#### 2.3.1 Particle size

The particle size and polydispersity index (PdI) were measured using a dynamic light scattering with a Litesizer™ 500 instrument (Anton Paar, Graz, Austria). To avoid multiple-particle effects, the dispersions were diluted to 0.4 % (w/v) with distilled water. Average particle size measurements were performed in triplicate at 25 °C, and the results were averaged. For the FCON and PCON, particle size was significantly larger and beyond the effective detection range of the Litesizer™ 500. Therefore, the particle size distribution (D_3,2_) and span of the control groups were measured using a laser diffraction particle size analyzer (Mastersizer 3000E, Malvern Panalytical, Worcestershire, UK). Measurements were performed in triplicate at 25 °C after dispersing the protein powder in distilled water and stirring to achieve a uniform suspension.

#### 2.3.2 ζ-potential

The ζ-potential of legume protein dispersions was measured using electrophoretic light scattering with a Litesizer™ 500 instrument (Anton Paar, Graz, Austria). The dispersions were diluted to 0.4 % (w/v) with distilled water. The ζ-potential of each sample was measured in triplicate at 25 °C.

#### 2.3.3 Solubility

Protein solubility, defined as the proportion of soluble protein relative to total protein content, was determined using a modified version of the method described by Yan et al. [25]. Protein dispersions were diluted to 1 % (w/v) with distilled water and centrifuged at 10,000 × g for 15 min at 4 °C. The protein concentration in the supernatant was quantified using the Biuret method, with absorbance measured at 540 nm using a UV–Vis spectrophotometer (Multiskan Go, Thermo Scientific™, Waltham, MA, USA). Solubility (%) was calculated using the following equation (1):

(1) $$Solubility\;\left(\%\right)=\frac{Protein\;concentraion\;in\;supernatant}{Initial\;protein\;concentration}\times 100$$

#### 2.3.4 Turbidity

Turbidity was assessed according to a modified version of the method described by Zhao et al. [26]. The samples were diluted to 1 % (w/v) in distilled water, and the absorbance was recorded at 600 nm using a UV–Vis spectrophotometer. Distilled water was used as the blank. Absorbance at 600 nm has been reported as a turbidity indicator that reflects the extent of light scattering by suspended protein particles.

#### 2.3.5 Visual appearance

The vials containing the samples were inverted and the external appearance of the samples was captured using a digital camera (α350, Sony, Tokyo, Japan).

#### 2.3.6 Optical microscopy of protein particles

Images were obtained using an optical microscope (CX 31; Olympus, Tokyo, Japan) coupled to a CCD camera (3.0 M, Olympus) at 400 × magnification. Prior to imaging, the particles were stained with Coomassie Brilliant Blue for enhanced visualization. All images were captured using a digital camera (Alpha 350; Sony, Tokyo, Japan).

#### 2.3.7 SDS-PAGE

Using a gel electrophoresis device (Bio-Rad Laboratories Ltd., Hercules, CA, USA), sodium dodecyl sulfate polyacrylamide gel electrophoresis (SDS-PAGE) was carried out using a discontinuous buffer system with 4 % stacking gel (pH 6.8) and 12 % separating gel (pH 8.8). A Laemmli sample buffer (Bio-Rad, Hercules, CA, USA) containing 5 % 2-mercaptoethanol was used to create protein samples under reducing conditions. The final protein concentration was 2 μg/μL.

#### 2.3.8 Surface hydrophobicity

Surface hydrophobicity (H_o_) was determined using 8-anilinonaphthalene-1-sulfonic acid (ANS) as a fluorescent probe based on the method described by Shao et al. [27] with slight modifications. Protein dispersions were prepared in phosphate buffer (pH 7.0) and serially diluted to obtain a range of protein concentrations. The ANS solution was added to each dilution, and the mixtures were incubated in the dark for 10 min 25 °C. The fluorescence intensity was measured using a spectrofluorophotometer (RF-5301 PC, Shimadzu, Kyoto, Japan) with an emission wavelength of 484 nm and an excitation wavelength of 390 nm for FBPIs and 365 nm for PPIs. The excitation and emission slit widths were both set to 3 nm. H_o_ was expressed as the initial slope of the fluorescence intensity versus protein concentration curve, calculated using linear regression and reported in relative fluorescence units (RFU).

#### 2.3.9 Fourier transform infrared spectroscopy

Fourier transform infrared spectroscopy (FT-IR) spectroscopy was performed to analyze the secondary structures of FBPIs and PPIs based on the method described by Choi et al. [23] with modifications. The protein dispersions were freeze-dried and finely ground to obtain a homogeneous powder. FT-IR spectra were acquired using a Tensor 27 spectrometer (Tensor 27, Bruker, Billerica, MA, USA) in transmittance mode, with a spectral resolution of 4 cm^−1^ and 32 accumulated scans over the range 600–4000 cm^−1^.

#### 2.3.10 Circular dichroism spectroscopy

The secondary structures of the legmue protein samples were analyzed using a circular dichroism spectrometer (Chirascan Plus, Applied Photophysics Ltd., Leatherhead, UK). Measurements were performed in the far-UV range (190–260 nm) using a quartz cuvette with a path length of 1 mm. Protein concentration was adjusted to 0.1 mg/mL. The resulting spectra were further deconvoluted to estimate the secondary structure composition using Circular Dichroism spectroscopy deconvolution software (CDNN).

### 2.4 Preparation of high internal phase Pickering emulsions (HIPPEs)

The HIPPEs were formulated using 4 % (w/v) FBPI or PPI dispersions as the continuous aqueous phase and canola oil as the dispersed phase, with an oil volume fraction of 70 % (w/v). The oil was slowly added to the protein dispersion under continuous stirring. Emulsification was performed using a high-speed homogenizer (T25 digital Ultra-turrax® high-speed mixer, IKA, Staufen, Germany) at 10,000 rpm for 5 min, while the mixture was maintained in an ice-water bath to prevent temperature rise during homogenization.

### 2.5 Droplet size

The droplet size and size distribution of the HIPPEs were measured using a laser diffraction particle size analyzer (Mastersizer 3000E, Malvern Panalytical, Worcestershire, UK). Each emulsion sample was diluted with distilled water to an obscuration level of approximately 7 %. The refractive indices were set to 1.465 for the oil phase (canola oil) and 1.330 for the aqueous phase (protein dispersion).

The volume-weighted mean diameter (D_4,3_) was used to represent the average droplet size, while the volume-based particle size distribution provided information on the droplet size uniformity. The span value was calculated according to equation (2):

(2) $$Span=\frac{D_{90}-D_{10}}{D_{50}}$$

was used to assess the width of the droplet size distribution, where D_10_, D_50_, and D_90_ are the diameters at 10 %, 50 %, and 90 % of the cumulative volume, respectively.

### 2.6 ζ- potential

The ζ-potential of HIPPEs was measured using electrophoretic light scattering with a Litesizer™ 500 instrument (Anton Paar, Graz, Austria). The emulsion samples were then diluted 40-fold with distilled water. A 1 mL aliquot of the diluted sample was transferred to a folded capillary cell, and the ζ-potential of each sample was measured in triplicate at 25 °C.

### 2.7 Optical microscopy of HIPPEs

Images were captured using an optical microscope (CX 31; Olympus, Tokyo, Japan) coupled with a CCD camera (3.0 M, Olympus) at 1,000 × magnification. photographed with a digital camera (Alpha 350; Sony, Tokyo, Japan).

### 2.8 Confocal laser scanning microscopy

The microstructures of the HIPPEs samples were visualized using a confocal laser scanning microscope (CLSM; LSM 800; Carl Zeiss, Oberkochen, Germany). Prior to imaging, each emulsion was diluted 5-fold with distilled water to reduce droplet crowding and facilitate clear visualization. The diluted emulsions were sequentially stained with Nile red and Nile blue A solutions, both at a concentration of 0.01 % (w/v), to label the oil and aqueous phases, respectively. Specifically, Nile red and Nile blue A were added to the diluted emulsions at final concentrations of approximately 25 μL/mL and 20 μL/mL, respectively, followed by gentle mixing to ensure homogeneous staining. A 5 μL aliquot of each diluted emulsion was mounted on a glass slide and covered with a cover slip. Fluorescence was detected at excitation wavelengths of 488 nm for Nile red (oil phase) and 633 nm for Nile blue A (aqueous phase).

### 2.9 Transmission electron microscopy

The interfacial microstructures of the HIPPEs were examined using a transmission electron microscope (TEM; JEM-1010, Jeol Ltd., Tokyo, Japan) operated at an acceleration voltage of 63 kV. Each HIPPE sample was diluted 300-fold with distilled water and adjusted to the same pH as that of the original formulation. A 10 μL aliquot of the diluted emulsion was deposited onto a 300-mesh copper grid and allowed to adsorb for 30 s. Excess liquid was removed using filter paper. For negative staining, 10 μL of 2 % (w/v) uranyl acetate solution was applied to the grid and removed after 30 s. The prepared grids were dried in a desiccator before imaging.

### 2.10 Adsorbed protein at interface and interfacial protein concentration

The adsorbed protein at interface (AP%) and interfacial protein concentration (*Γ*) of HIPPEs was determined based on the method of Choi et al. and Shao and Tang [23,28] with slight modifications. HIPPE samples were centrifuged at 10,000 × g for 30 min at 4 °C to separate the serum phase. The aqueous serum layer was carefully withdrawn using a syringe and filtered through a 0.45 μm membrane filter (ADVANTEC, Tokyo, Japan). The protein concentration in the filtrate (C_f_) was determined using the biuret method. The initial protein concentration (C_o_) in the FBPI or PPI dispersions prior to emulsification was measured using a biuret assay. The AP and *Γ* was calculated using the following equation ((3), (4)):

(3) $$AP\;\left(\%\right)=\frac{\left(C_{o}-C_{f}\right)}{C_{o}}\times 100$$

(4) $$\Gamma \;\left(mg/m^{2}\right)=\frac{\left(C_{o}-C_{f}\right)\times D_{3,2}}{6ø}$$

D_3,2_ and ø are the surface average diameter and oil fraction (0.7), respectively.

### 2.11 Rheological properties

The rheological behavior of the HIPPEs was evaluated using a Rheometer® (MCR302, Anton Paar, Graz, Austria) equipped with a 25 mm parallel-plate geometry. Each emulsion sample was gently loaded onto the lower plate, and the upper plate was set with a fixed gap of 1.0 mm. The excess samples at the edges were carefully trimmed to ensure uniform contact. All measurements were conducted at 25 °C.

The apparent viscosities of HIPPEs were measured by varying the shear rate from 0.1 to 100 1/s. Subsequently, an amplitude sweep test was performed at a constant angular frequency of 10 rad/s with a strain ranging from 0.01 % to 100 % to determine the linear viscoelastic region (LVR). Within the LVR, a frequency sweep test was carried out to measure the storage modulus (G′) and loss modulus (G″), which represent the elastic and viscous behaviors of the emulsions, respectively.

### 2.12 Statistical analysis

All reported values were derived from testing in triplicate (or more), and results are presented as mean ± standard deviation of at least three experiments. Means were compared using one-way ANOVA followed by Duncan’s multiple range test (*p* < 0.05). Statistical analyses were performed using the SPSS software (version 24.0; SPCC Inc., Chicago, IL, USA).

## 3 Results and discussion

### 3.1 Physicochemical changes in FBPIs and PPIs by heat-ultrasound treatment

#### 3.1.1 Particle size and surface charge

**Fig. 1:**
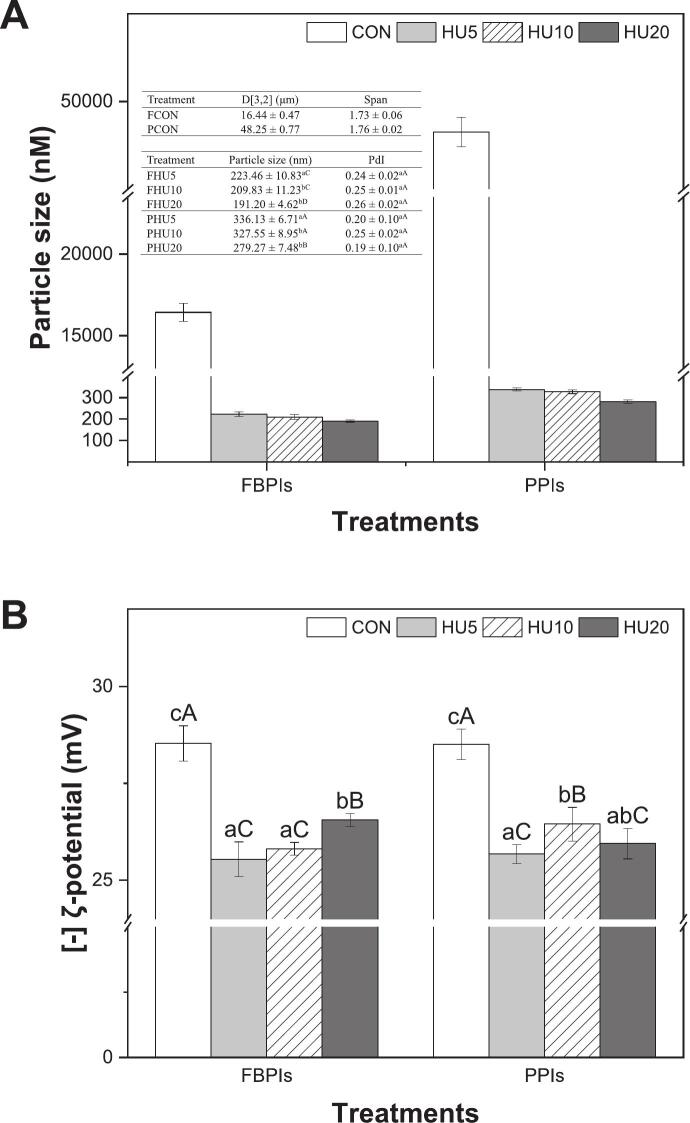
Particle size (A) and ζ-potential (B) of HU-treated legume protein isolates. FBPIs, faba bean protein isolates; PPIs, pea protein isolates; HU, 4 % protein dispersions sequentially heated and ultrasonicated for 5, 10 and 20 min, respectively. ^a–b^ Data labeled with different lowercase letters indicate a significant difference among treatments within each legume protein group (*p* < 0.05). ^A–C^ Data labeled with different uppercase letters indicate a significant difference across all treatments (*p* < 0.05).

The physicochemical properties of protein particles, particularly their size and surface charge, play an important role in their behavior at the oil–water interface in Pickering emulsions. Smaller particles provide higher surface areas and faster diffusivity, allowing for more effective interfacial coverage and improved steric stabilization. Simultaneously, sufficient surface charge, as indicated by ζ-potential, helps maintain dispersion stability through electrostatic repulsion [2,6]. As shown in Fig. 1, the sequential applications of HU significantly altered both particles size and ζ-potential in FBPIs and PPIs. Untreated samples (FCON and PCON) exhibited large aggregated particles, with diameter of 16.44 μm and 48.25 μm, respectively (Fig. 1A). These aggregates are commonly observed in spray-dried protein isolates due to partial denaturation and intermolecular crosslinking during industrial processing [29,30].

Following HU treatments, particle size decreased dramatically, with FBPI reducing from 223.5 nm (HU5) to 191.2 nm (HU20), and PPI from 336.1 nm (HU5) to 279.3 nm (HU20) (*p* < 0.05). This size reduction reflects the thermal unfolding and ultrasonic cavitation effects, which disrupt the aggregated structures and enhance dispersion. In both protein groups, heat treatment likely caused partial unfolding of the protein structures, exposing hydrophobic regions and weakening intra- and intermolecular bonds. Subsequent ultrasonication generates cavitation bubbles and high shear forces that physically fragment the unfolded protein aggregates, resulting in smaller and more dispersed particles [31]. As the duration of ultrasonication increased from 5 to 20 min, a progressive decrease in particle size was observed for both protein isolates. This size reduction was more pronounced in FBPIs, likely because of its more flexible globular structure, which made it more susceptible to disassembly under shear. These changes suggest that the FBPI particles may pack more efficiently at the interface and form more stable films in HIPPEs than in PPIs.

In parallel, ζ-potential values decreased significantly upon HU treatment (Fig. 1B). At pH 7.0, all the samples exhibited negative surface charges owing to the deprotonation of the acidic side chains. The pIs of both FBPIs and PPIs have been reported to be in the range of approximately pH 4.3–4.8 [32,33]. At pH values above the pI, the deprotonation of carboxyl groups leads to the predominance of negatively charged –COO- moieties, resulting in a net negative ζ-potential [34]. FCON and PCON showed the highest absolute values (about 28.50 mV), while HU-treated particles ranged from 26.65 to 25.54 mV for FBPIs and from 26.63 to 25.68 mV for PPIs. This decrease in absolute ζ-potential could be explained by structural rearrangements induced by the treatments. Ultrasonication has been reported to induce the unfolding of secondary structures and increase the exposure of nonpolar hydrophobic residues, which may reduce the number of negatively charged groups present at the particle surface, contributing to the observed decline in surface charge [35]. According to [36], high intensity ultrasonication treatment decreases the negative surface charge on proteins, which decreases the electrostatic barrier and increases the adsorption rate, thus contributing to foaming properties. This may also result from high-intensity ultrasonication promoting aggregate formation via electrostatic interactions and reducing repulsion and surface charge [37].

#### 3.1.2 Solubility and turbidity

**Fig. 2:**
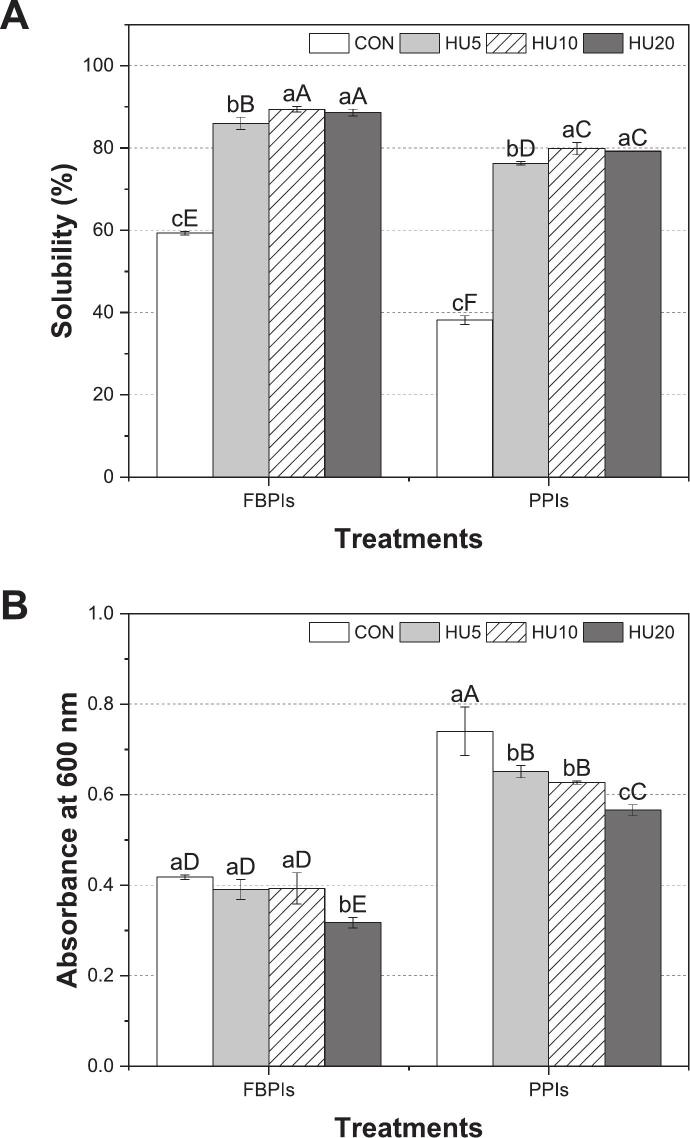
Solubility (A) and turbidity (B) of HU-treated legume protein isolates. FBPIs, faba bean protein isolates; PPIs, pea protein isolates; CON, untreated 4 % protein dispersions; HU, 4 % protein dispersions sequentially heated and ultrasonicated for 5, 10 and 20 min, respectively. ^a–c^ Data labeled with different lowercase letters indicate a statistically significant difference among treatments within each legume protein group (*p* < 0.05). ^A–F^ Data labeled with different uppercase letters indicate a significant difference across all samples (*p* < 0.05).

Solubility and turbidity are critical indicators of protein dispersion behavior in aqueous media and are closely linked to the particle size, aggregate structure, and interfacial potential in emulsion systems. In this study, HU treatment significantly improved the solubility of both FBPIs and PPIs (Fig. 2A) while reducing turbidity through the disaggregation of protein clusters (Fig. 2B).

In both protein groups, the untreated samples exhibited the lowest solubility (59.29 % in FCON and 38.16 % in PCON, *p* < 0.05). As previously discussed, industrial procedures such as spray drying tend to produce larger aggregated protein particles due to partial denaturation and aggregation, which hinders dispersion and reduces interfacial functionality, ultimately reducing solubility [29,30]. Between the two protein isolates, FBPIs consistently exhibited higher solubility than PPIs across all treatments (*p* < 0.05), likely because of differences in globulin composition. Legume proteins are primarily composed of vicilin (7S) and legumin (11S), which differ in molecular structure and polarity and strongly influence protein solubility [22]. FBPIs generally contain a higher proportion of 7S, whereas PPIs are more abundant in 11S. This compositional distinction is reflected in the reported 11S/7S ratios, which vary widely among pea genotypes, ranging from less than 1 to as high as 8.7, indicating the strong dominance of legumin in certain cultivars [17,20,38]. Building on this compositional difference, the structural characteristics of 7S and 11S globulins explain their different solubility profiles. The lower molecular weight, higher polarity, and fewer disulfide bonds of 7S contributed to its better solubility and greater conformational flexibility, whereas the rigid disulfide-stabilized structure of 11S tended to aggregate and disperse poorly in aqueous systems.

To further elucidate the effects of HU treatment, a significant increase in solubility was observed, reaching 89.42 % in FHU10 and 79.85 % in PHU10 (*p* < 0.05). Heat treatment partially unfolds protein structures, exposing buried hydrophilic groups that improve their interactions with water molecules [21]. In addition, high-intensity ultrasound-induced cavitation causes conformational rearrangements that reorient the internal hydrophilic residues towards the aqueous phase, enhancing solubility [39]. Furthermore, the reduction of particle size (Fig. 1A) to the nanoscale enhances mobility and dispersion, further contributing to solubility [40]. The present results are in line with those of [10], who demonstrated that the sequential application of heat treatment followed by ultrasonication effectively altered the structure of rice protein isolate, leading to enhanced solubility. These improvements have been attributed to protein unfolding, disruption of non-covalent interactions, and disaggregation of protein complexes induced by combined thermal and mechanical effects. Within each protein group, the solubility increased progressively with increasing ultrasonication time. However, no statistically significant difference was observed between the 10 and 20 min treatments, suggesting that the extent of structural modification required for solubility enhancement was largely achieved after 10 min of ultrasonication.

**Fig. 3:**
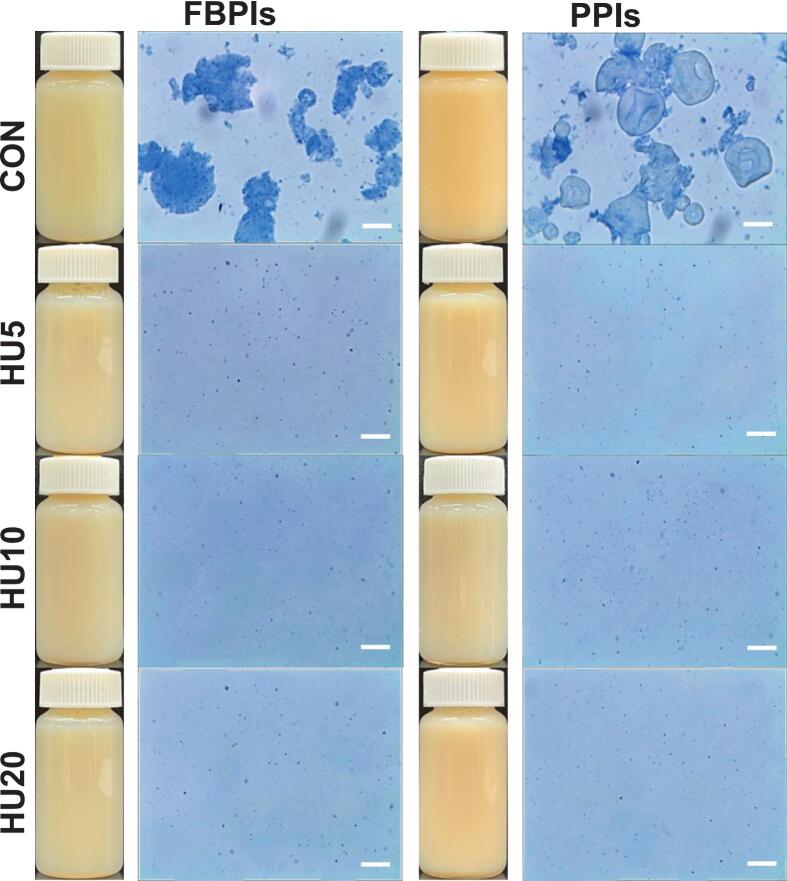
Visual appearance and optical microscopic images of 4 % protein dispersions of HU-treated legume protein isolates. FBPIs, faba bean protein isolates; PPIs, pea protein isolates; CON, untreated 4 % protein dispersions; HU, 4 % protein dispersions sequentially heated and ultrasonicated for 5, 10 and 20 min respectively. Scale bar = 40 μm.

Turbidity analysis was conducted to evaluate the light scattering in the protein dispersions, which is primarily influenced by the size and solubility of the suspended particles [24]. In general, turbidity is directly proportional to the particle size, with larger aggregates scattering more light and yielding higher absorbance values [23]. As shown in Fig. 3, both visual inspection and optical microscopy were employed in parallel with turbidity measurements (Fig. 2B) to qualitatively support the detection of protein aggregation and observe structural changes.

FCON and PCON showed the highest turbidity within each group (*p* < 0.05), which was consistent with the large irregular particles observed by optical microscopy. These microscopic images revealed dense, aggregated protein structures in the CON samples, which visibly diminished in size (Fig. 1A) and uniformity after HU treatment. Upon HU treatment, the turbidity decreased in both protein groups, indicating improved dispersion and reduced aggregate size. However, the extent and rate of turbidity reduction differed among proteins. For PPIs, turbidity decreased progressively with increasing sonication time (*p* < 0.05), whereas FBPI required at least 10 min of sonication before showing a statistically significant change. This suggests that PPIs aggregates are more readily disrupted under ultrasound, despite their overall lower solubility, whereas the more stable globular structure of FBPIs resists disintegration without prolonged energy input. These findings are in line with those of previous studies reporting that controlled heat and sonication can disrupt protein-protein interactions, enhance structural stability, and reduce aggregate size and turbidity [41], which aligns with the observed decrease in particle size following treatment. Additionally, [42] demonstrated that ultrasonic cavitation and mechanical shear facilitated the breakdown of large aggregates into smaller, more soluble particles, contributing to reduced turbidity.

#### 3.1.3 SDS-PAGE

**Fig. 4:**
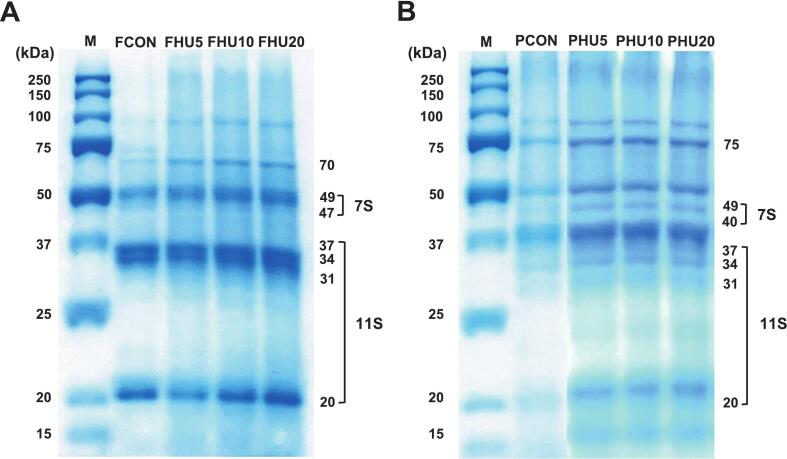
SDS-PAGE of HU-treated legume protein isolates. F, faba bean protein isolates; P, pea protein isolates; CON, untreated 4 % protein dispersions; HU, 4 % protein dispersions sequentially heated and ultrasonicated for 5, 10 and 20 min, respectively.

To determine whether the HU treatment altered the primary structure of legume proteins, SDS-PAGE analysis was performed on FBPIs and PPIs (Fig. 4). No notable changes in molecular weight distribution were observed between the untreated (FCON, PCON) and HU-treated samples. The major protein bands remained at similar positions across all treatments, indicating that HU treatment did not cleave the peptide bonds or disrupt the covalent backbone of the proteins. This observation is consistent with previous reports showing that moderate ultrasound does not affect the primary structure of proteins, as peptide bonds remain intact [43,44].

While the banding patterns remained constant, distinct differences in band intensities were evident. FCON and PCON exhibited visibly weaker bands than their HU-treated counterparts, despite equal protein loading across lanes. This reduced intensity likely reflects the lower solubility of the untreated proteins, which limits protein extraction and SDS-mediated denaturation during sample preparation. This interpretation was supported by the solubility data (Fig. 2A), in which HU treatment significantly improved the solubility of both FBPIs and PPIs. Previous studies have shown that enhanced solubilization facilitates better band resolution in SDS-PAGE [45].

The presence of legume globulin subunits in both the FBPI and PPI was confirmed. Bands corresponding to 50–60 kDa (vicilin, 7S) and 40–20 kDa (acidic and basic subunits of legumin, 11S) were observed, which is consistent with the known profiles of legume storage proteins [17,46]. Although the qualitative band patterns were similar, the improvement in extractability and visibility after HU treatment indirectly highlighted the structural loosening and dispersion of protein aggregates, as observed in the solubility (Fig. 2A) and particle size analyses (Fig. 1A). These results support the conclusion that HU treatment facilitates efficient protein mobilization without compromising the integrity of the primary structure.

#### 3.1.4 Surface hydrophobicity

Surface hydrophobicity (H_o_) is a critical structural parameter that directly influences the functional properties of proteins, particularly their emulsifying and foaming capacities. This reflects the extent to which hydrophobic amino acid residues are exposed to the aqueous environment, thereby facilitating adsorption at the oil–water interface and the formation of a stable interfacial layer [47].

**Fig. 5:**
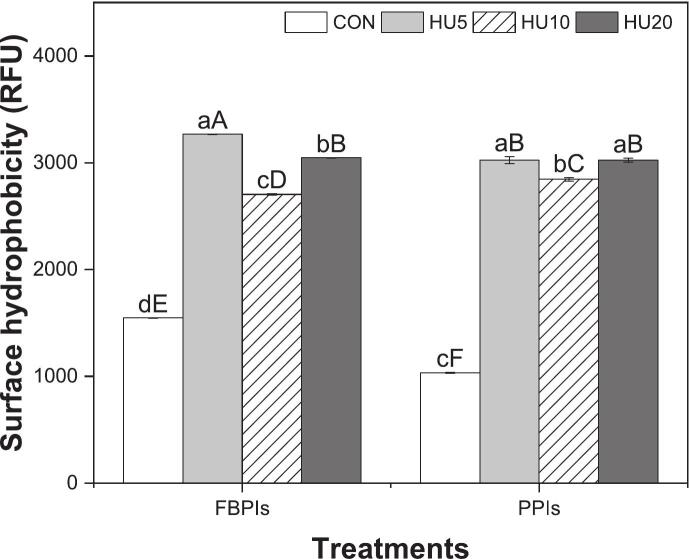
Surface hydrophobicity of HU-treated legume protein isolates. FBPIs, faba bean protein isolates; PPIs, pea protein isolates; CON, untreated 4 % protein dispersions; HU, 4 % protein dispersions sequentially heated and ultrasonicated for 5, 10 and 20 min, respectively. ^a–d^ Data labeled with different lowercase letters indicate a significant difference among treatments within each legume protein group (*p* < 0.05). ^A–F^ Data labeled with different uppercase letters indicate a significant difference across all samples (*p* < 0.05).

As shown in Fig. 5, HU treatment significantly increased the H_o_ levels in both FBPIs and PPIs (*p* < 0.05). FBPI values rose from 1547.45 to 3267.70 RFU, and PPI values increased from 1033.55 to 3024.85 RFU, representing more than a twofold increase in both cases. This can be attributed to partial unfolding by heat, which exposes buried hydrophobic residues, and further structural disassembly induced by sonication, which fragments, aggregates, and redistributes the hydrophobic regions originally embedded within the soluble protein structures [48,49]. Between the two proteins, FBPIs not only started from a higher baseline but also exhibited more pronounced and time-responsive variations in H_o_ across the HU treatments, suggesting greater structural adaptability and sensitivity to cavitation-induced modification compared to PPIs.

Interestingly, H_o_ did not increase linearly with the ultrasonication time. In both protein groups, the highest H_o_ was observed at 5 min (HU5), followed by a significant decrease at 10 min (HU10), and partial recovery at 20 min (HU20) (*p* < 0.05). These results suggest that moderate sonication for 5 min optimally disrupts aggregates and exposes hydrophobic regions, whereas prolonged treatment (10 min) may induce partial reaggregation or structural rearrangement via hydrophobic interactions, reducing the availability of hydrophobic groups on the surface. [50] reported that an increased ultrasound processing time may cause partial denaturation of protein structures, followed by repolymerization through hydrophobic interactions and other mechanisms, ultimately leading to a decrease in H_o_.

However, a complementary interpretation is possible. According to [37], the H_o_ of PPIs initially increased following ultrasound treatment but decreased with prolonged exposure yet remained higher than native samples. This behavior closely resembles the pattern observed in the present study, where the formation of more extended soluble aggregates may increase the overall surface area, but does not necessarily lead to greater exposure of hydrophobic residues, thereby reducing the number of effective binding sites for ANS probes. At 20 min, partial recovery of H_o_ was observed, indicating that extended ultrasound treatment may have disrupted the previously formed aggregates or further unfolded protein domains, thereby re-exposing the buried hydrophobic sites. A similar pattern was observed by [51], in which initial sonication unfolded proteins and exposed hydrophobic regions, whereas prolonged treatment caused aggregation and reduced H_o_, followed by the re-exposure of hydrophobic sites upon extended sonication. However, H_o_ at HU20 did not return to the level observed at HU5, implying that some aggregation or structural rearrangement during the intermediate treatment (HU10) may be partially irreversible. Thus, while further sonication can recover hydrophobic exposure to some extent, the initial conformation induced by moderate sonication appears to be the most favorable for maximizing H_o._

Notably, changes in H_o_ closely aligned with solubility behavior (Fig. 2A), suggesting that increased exposure to both hydrophobic and hydrophilic residues reduce protein-protein interactions and improves dispersibility [51]. This relationship was also reported by [50], [23], where increased H_o_ due to protein unfolding was accompanied by higher solubility in soy and mung bean protein isolates.

#### 3.1.5 Fourier transform infrared spectroscopy

Fourier-transform infrared spectroscopy (FT-IR) was used to investigate secondary structural changes in FBPIs and PPIs induced by HU treatment. Spectral regions were analyzed, with particular focus on the Amide I band (1700–1600 cm^−1^), which is primarily associated with C=O stretching vibrations and is sensitive to protein secondary structure such as α-helix and β-sheet. Additional bands include the Amide II (1580–1480 cm^−1^), associated with N–H bending and C–N stretching, while the Amide A band (3400–3200 cm^−1^) is attributed to N–H stretching and hydrogen bonding. The CH_2_ stretching band (∼2930 cm^−1^) provides additional information on hydrophobic chain environments.

**Fig. 6:**
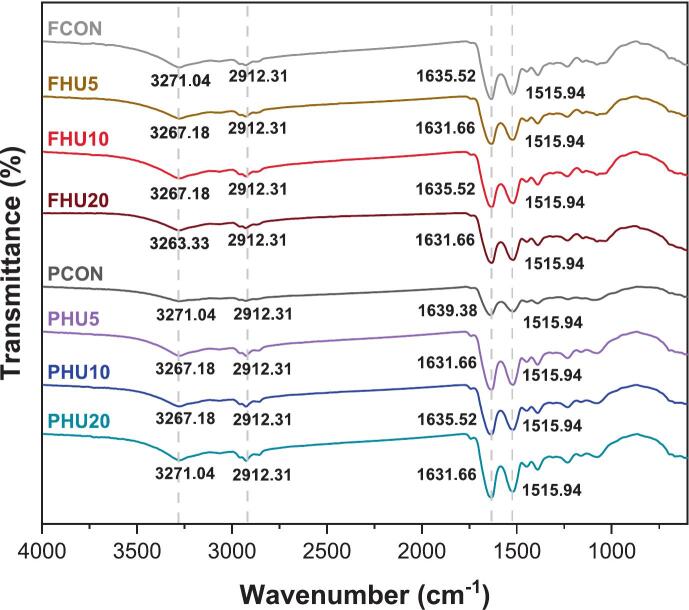
FT-IR spectra of HU-treated legume protein isolates. F, faba bean protein isolates; P, pea protein isolates; CON, untreated 4 % protein dispersions; HU, 4 % protein dispersions sequentially heated and ultrasonicated for 5, 10 and 20 min, respectively.

As shown in Fig. 6, the Amide I band in the untreated samples of FCON and PCON appeared at 1635.52 and 1639.38 cm^−1^, respectively, whereas the HU-treated samples exhibited a redshift to 1631.66–1635.52 cm^−1^. Similar downshifts were observed in the Amide A region, where the control peak moved from 3271.04 cm^−1^ to 3263.33^−^3267.18 cm^−1^ after treatment. Such redshifts in the Amide I region can be attributed to increased hydrogen bonding and rearrangement of the backbone C=O groups, suggesting partial unfolding and reorganization of protein secondary structure [52].

These observations are consistent with previous studies, where redshifts in the Amide I band have been associated with conformational transitions, particularly changes in β-sheet and α-helix content [53], [54]. However, because of the overlapping nature of FT-IR bands, precise quantification of secondary structure components remains limited. To complement this, circular dichroism (CD) spectroscopy was employed, providing more accurate assessment of conformational changes. CD results confirmed an increase in α-helix content and a reduction in random coil and β-turn structures, consistent with the FT-IR-based interpretation of partial unfolding and reorganization upon HU treatment.

#### 3.1.6 Circular dichroism spectroscopy

Circular dichroism spectroscopy (CD) was used to evaluate secondary structural changes in FBPIs and PPIs after HU treatment. As shown in Table 1, both protein groups showed a marked increase in α-helix content and a corresponding reduction in β-sheet, β-turn, and random coil structures compared to the untreated control (*p* < 0.05). These results suggested that HU treatment induces partial unfolding, followed by molecular reorganization into a more ordered conformation [55]. For FBPIs, α-helix content increased from 23.90 % to 35.90 % (*p* < 0.05), while PPIs showed a more dramatic shift from a lower baseline of 7.90 % to 29.85 %. This difference indicates that although both proteins are responsive to HU treatment, FBPIs exhibit greater conformational flexibility and structural adaptability under thermal and mechanical stress.

**Table 1:** Secondary structural content of HU-treated legume protein isolates estimated from CD spectra.

Treatment	α-helix (%)	β-sheet (%)	Random coil (%)	β-turn (%)
FCON	23.90 ± 0.90^cE^	22.20 ± 0.40^aB^	46.35 ± 0.15^aB^	12.20 ± 0.10^aB^
FHU5	32.75 ± 0.65^bB^	21.10 ± 0.30^bCD^	41.00 ± 0.40^bC^	5.70 ± 0.30^bF^
FHU10	35.90 ± 0.40^aA^	20.50 ± 0.10^bDE^	38.45 ± 0.65^cD^	3.45 ± 0.05^dH^
FHU20	33.25 ± 0.45^bB^	17.50 ± 0.60^cF^	40.65 ± 0.25^bC^	5.00 ± 0.10^cG^
PCON	7.90 ± 0.10^cF^	27.00 ± 0.10^aA^	49.70 ± 0.00^aA^	15.40 ± 0.00^aA^
PHU5	28.70 ± 0.30^bD^	21.60 ± 0.80^bBC^	41.25 ± 0.95^bC^	8.45 ± 0.15^cD^
PHU10	29.85 ± 0.75^aC^	20.55 ± 0.15^cDE^	41.50 ± 0.80^bC^	8.10 ± 0.20^dE^
PHU20	29.45 ± 0.15^abCD^	20.20 ± 0.00^cDE^	41.05 ± 0.05^bC^	9.35 ± 0.05^bC^

F, faba bean protein isolates; P, pea protein isolates; CON, untreated 4 % protein dispersions; HU, 4 % protein dispersions sequentially heated and ultrasonicated for 5, 10 and 20 min, respectively. ^a–d^ Data labeled with different lowercase letters within each column indicate a significant difference among treatments within the same legume protein group (*p* < 0.05). ^A–^^H^ Data labeled with different uppercase letters within each column indicate a significant difference across all treatments (*p* < 0.05).

Interestingly, although α-helix content is often reported to decrease under thermal or ultrasonic treatment due to protein denaturation [54], [56], the present findings suggest that moderate energy input can facilitate partial unfolding followed by refolding into stable helical structures. Similar patterns have been reported by [57] showing that thermal treatment and ultrasound waves increased α-helix and reduced β-sheet and turn content of milk proteins, possibly due to refolding of partially unfolded chains into more ordered helices under moderate shear stress. Similarly, [44] observed an increase in α-helix and a decrease in β-sheet and β-turn content in glycinin subjected to mild heating and ultrasound treatments, suggesting these treatments disrupt and weaken the intermolecular interactions of protein. The concurrent decrease in β-sheet, β-turn, and random coil structures further supports the formation of more ordered conformations.

Crucially, the CD results directly complemented the H_o_ measurements. HU treatment promoted both the exposure of buried hydrophobic residues and the refolding of backbones into α-helix rich conformations. The exposed hydrophobic patches enhanced anchoring at the oil–water interface, while the enriched α-helix content enhanced conformational flexibility, enabling efficient molecular rearrange and interconnection. Thus, these structural changes are expected to facilitate the formation of cohesive and elastic interfacial films, a relationship further demonstrated in the following section on HIPPEs stability.

### 3.2 Pickering emulsion stabilization by heat-ultrasound modified FBPIs and PPIs

#### 3.2.1 Macroscopic and colloidal features of HIPPEs

In Pickering emulsions, the droplet size plays a critical role in determining the physical stability. Smaller particles can diffuse more rapidly and adsorb more effectively at the oil–water interface, resulting in finer droplets and enhanced emulsion stability [2]. As shown in Table 2, HIPPEs stabilized by HU-treated protein particles exhibited significantly smaller droplet sizes (*p* < 0.05) than those stabilized by untreated controls. This observation was consistent with the particle size reduction (Fig. 1A), indicating that physical modification of the protein facilitated more efficient emulsification. Interestingly, the smallest droplet sizes were observed in the HU10 treatments for both FBPIs and PPIs, although the HU20 samples had slightly smaller particle sizes (*p* < 0.05). These findings imply that beyond particle size, other interfacial properties, such as solubility and H_o_, might also contribute to the regulation of Pickering emulsions.

**Table 2:** Droplet size (D_4,3_), span value, and ζ-potential of HIPPEs stabilized by HU-treated legume protein isolates.

Treatment	D_4,3_ (μm)		Span		[-] ζ-potential (mV)
FCON	15.12 ± 0.45^abBC^		2.22 ± 0.09^A^		45.68 ± 0.92^cB^
FHU5	14.28 ± 0.45^bC^		2.08 ± 0.08^abA^		37.13 ± 1.34^abC^
FHU10	14.36 ± 1.10^bC^		2.02 ± 0.28^bA^		37.15 ± 0.79^cC^
FHU20	16.10 ± 0.57^a^^B^		2.08 ± 0.10^abA^		35.67 ± 0.28^a^^C^
PCON	26.19 ± 0.59^a^^A^		2.13 ± 0.07^a^^A^		50.92 ± 2.77^bA^
PHU5	16.33 ± 0.66^bB^		1.77 ± 0.04^bB^		46.21 ± 0.40^a^^B^
PHU10	11.63 ± 0.89^cD^		1.68 ± 0.12^bcB^		44.70 ± 1.25^a^^B^
PHU20	15.10 ± 1.51^bBC^		1.63 ± 0.06^cB^		45.98 ± 1.38^a^^B^

^a-c^ Data labeled with different lowercase letters within each column indicate a significant difference among treatments within each legume protein group (*p* < 0.05). ^A–D^ Data labeled with different uppercase letters within each column indicate a significant difference across all treatments (*p* < 0.05).

HIPPEs, high internal phase Pickering emulsion; F, faba bean protein isolates; P, pea protein isolates; CON, untreated 4 % protein dispersions; HU, 4 % protein dispersions sequentially heated and ultrasonicated for 5, 10 and 20 min, respectively.

**Fig. 7:**
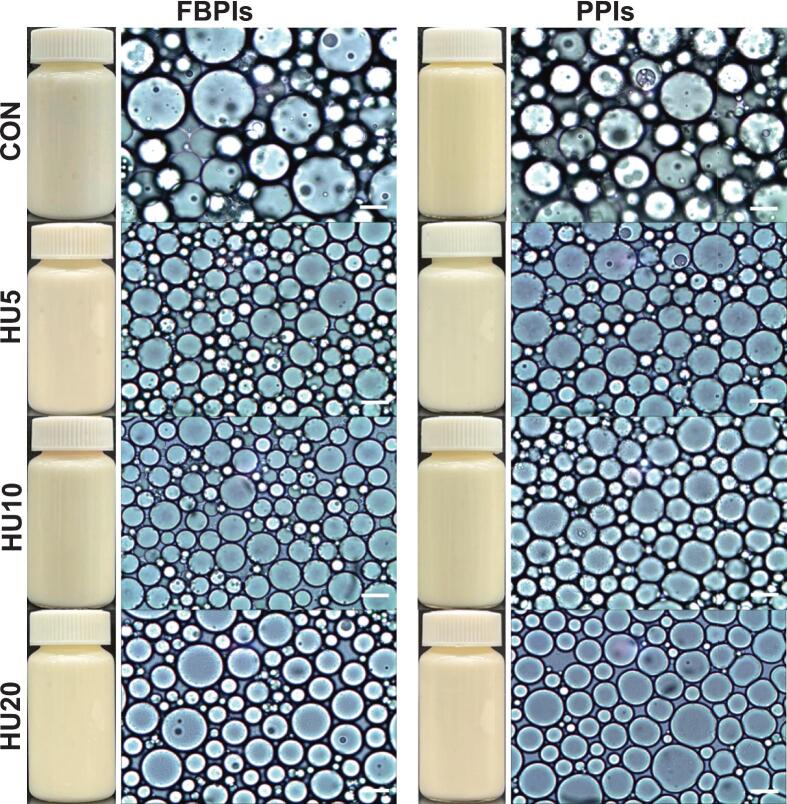
Visual appearance including morphology of HIPPEs stabilized by HU-treated legume protein isolates. HIPPEs, high internal phase Pickering emulsion; FBPIs, faba bean protein isolates; PPIs, pea protein isolates; CON, untreated 4 % protein dispersions; HU, 4 % protein dispersions sequentially heated and ultrasonicated for 5, 10 and 20 min, respectively. Scale bar = 20 μm.

**Fig. 8:**
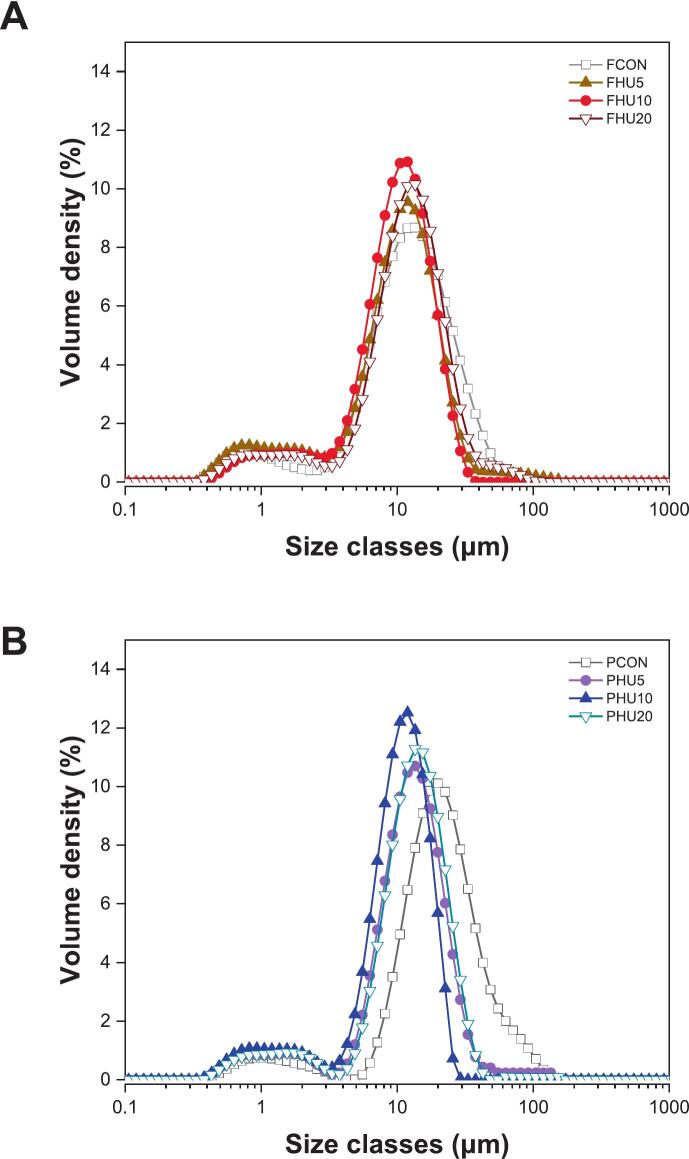
Droplet size distribution of HIPPEs stabilized by HU-treated legume protein isolates. HIPPEs, high internal phase Pickering emulsion; F, faba bean protein isolates; P, pea protein isolates; CON, untreated 4 % protein dispersions; HU, 4 % protein dispersions sequentially heated and ultrasonicated for 5, 10 and 20 min, respectively.

Notably, the HU10 samples exhibited the lowest H_o_ (*p* < 0.05) among the treated groups (Fig. 5), whereas the HU20 samples showed increased H_o_, which coincided with a slight increase in droplet size. This implies that an excessively high H_o_ may hinder particle dispersibility and interfacial mobility, ultimately limiting effective coverage at the oil–water interface despite the advantage of smaller particle size. This phenomenon is supported by [48] who reported that a moderate level of H_o_ facilitates the spontaneous adsorption of protein particles, whereas overly hydrophobic surfaces can lead to aggregation in aqueous environments and reduced interfacial activity. Supporting this, Fig. 7, Fig. 8 show that the untreated samples formed large irregular droplets with poor dispersion, whereas the HIPPEs stabilized by HU-treated proteins exhibited smaller and more uniformly distributed droplets. The droplet size distribution curves of the HU-treated groups also shifted towards smaller sizes, reinforcing the conclusion that physical modification improved protein interfacial behavior and enhanced emulsion stabilization.

In line with this understanding, the surface charge is another important factor governing emulsion stability in Pickering systems. Protein particles with a sufficiently high surface charge can generate strong electrostatic repulsion, which prevents aggregation and facilitates the formation of a stable, densely packed monolayer at the oil–water interface, thereby enhancing emulsion stability [2].

Table 2 shows that all emulsions possessed negative ζ-potential values exceeding 30 mV, indicating sufficient electrostatic stabilization [58]. Among untreated samples, PCON-stabilized HIPPEs displayed a higher absolute ζ-potential (50.92 mV) (*p* < 0.05) than FCON (45.68 mV), suggesting a stronger electrostatic barrier between droplets in the PPI group. Following HU treatment, the absolute ζ-potential values decreased significantly in both protein groups down to 35.67 mV for FBPIs and 44.70 mV for PPIs (*p* < 0.05). This reduction likely attributed to structural unfolding and increased exposure of hydrophobic residues, which may shield or displace negatively charged groups on the protein surface [35]. Despite the decrease, the absolute ζ-potential values remained above 30 mV, indicating that sufficient surface charge was retained to ensure electrostatic stabilization of the emulsions. Given these findings, it is plausible that changes in surface charge of particles (Fig. 1B) contributed not only to colloidal stability but also influenced interfacial behavior. The reduction in surface charge following HU treatment may have affected the formation of interfacial films by altering electrostatic interactions between droplets. These results underscore the critical role of protein surface characteristics, including charge distribution and hydrophobicity, in determining the stability of high internal phase Pickering emulsions.

#### 3.2.2 Microscopic characterization of interfacial adsorption in HIPPEs

**Fig. 9:**
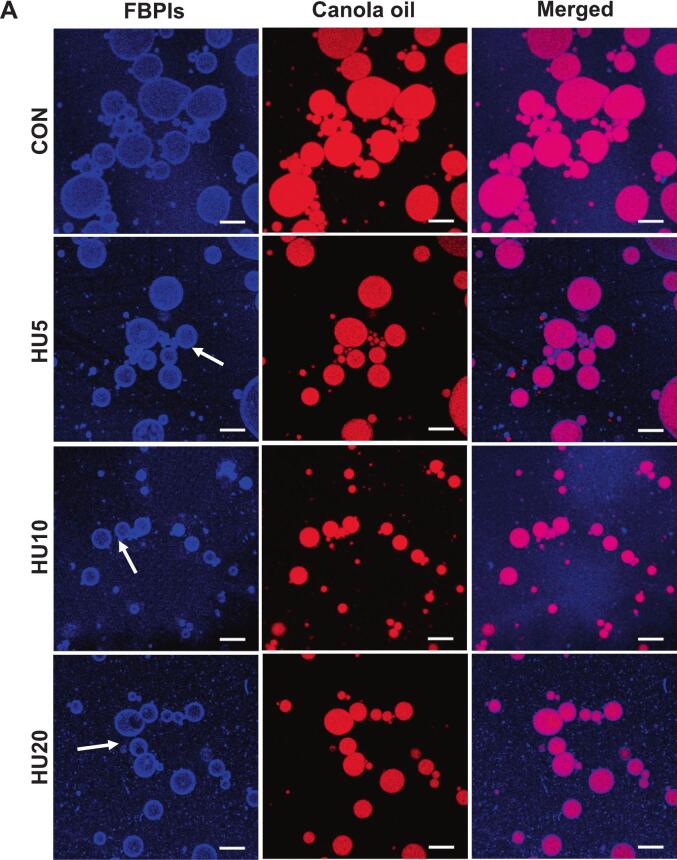

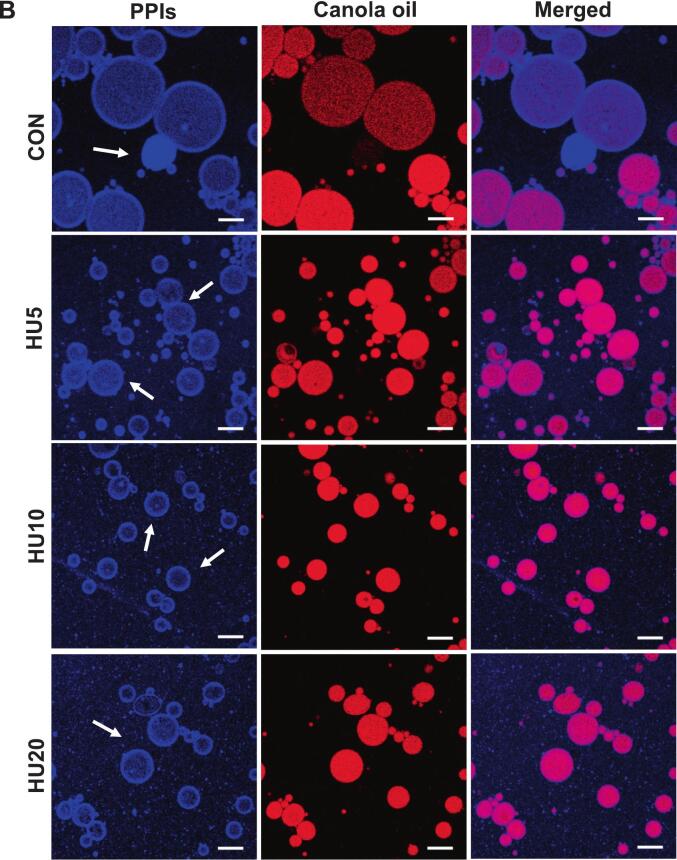
CLSM images of HIPPEs stabilized by HU-treated legume protein isolates, FBPIs (A) and PPIs (B). HIPPEs, high internal phase Pickering emulsion; FBPIs, faba bean protein isolates; PPIs, pea protein isolates; CON, untreated 4 % protein dispersions; HU, 4 % protein dispersions sequentially heated and ultrasonicated for 5, 10 and 20 min, respectively. Scale bar = 20 μm.

Confocal laser scanning microscopy (CLSM) was employed to visualize the adsorption behavior of protein particles at the oil–water interface. Proteins were stained with Nile Blue, and the oil phase was stained with Nile Red, enabling clear differentiation of the two phases. As shown in Fig. 9, the CLSM images revealed that in all treatments, protein particles were adsorbed at the oil–water interface, forming continuous interfacial layers, which is a defining characteristic of Pickering emulsions. This confirmed that the HU-treated FBPIs and PPIs acted as particulate emulsifiers capable of stabilizing droplets through interfacial adsorption. Consistent with the droplet size and optical microscopy data, the control emulsions (FCON and PCON) exhibited larger oil droplets and less uniform interfacial coverage. Notably, the PCON samples displayed large protein particle aggregates loosely adsorbed on the droplet surfaces (Fig. 9A), which agreed with the larger particle size observed for the PPIs in Fig. 1 A (48.25 μm). These observations suggest that, without physical modification, protein aggregates remain too large to effectively stabilize the interface.

**Fig. 10:**
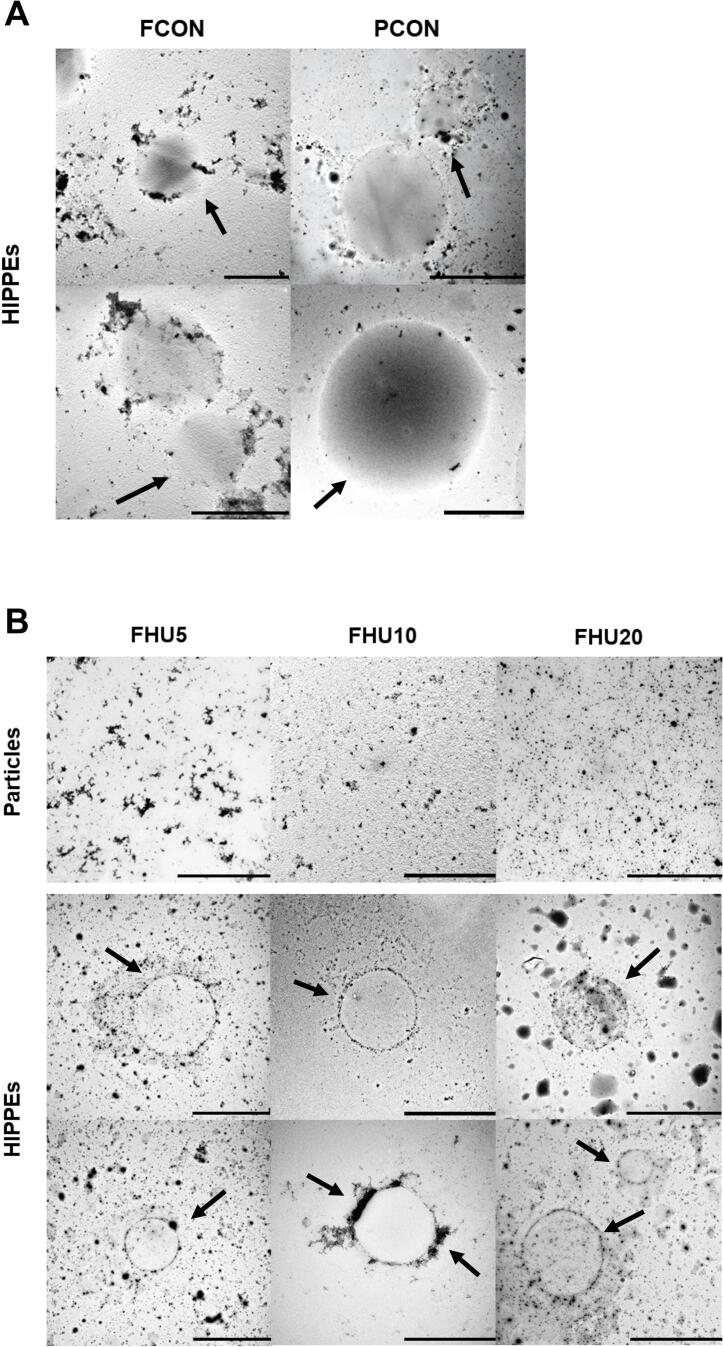

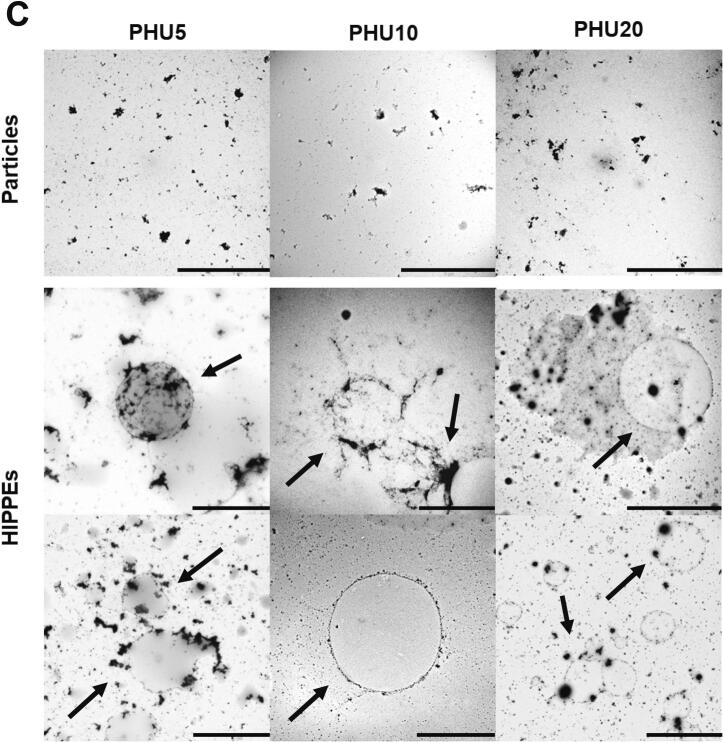
TEM images of legume protein particles and HIPPEs. HIPPEs stabilized by untreated protein isolates (FCON and PCON) (A), HU-treated FBPI particles and the corresponding HIPPEs (B), and HU-treated PPI particles and the corresponding HIPPEs (C). HIPPE, high internal phase Pickering emulsion; FBPIs (F), faba bean protein isolates; PPIs (P), pea protein isolates; CON, untreated 4 % protein dispersions; HU10, 4 % protein dispersions sequentially heated and ultrasonicated for 10 min; Scale bar = 10 μm.

To further examine the interfacial microstructure at higher resolution, transmission electron microscopy (TEM) was used (Fig. 10). In the TEM images, protein particles appear as dark regions because of their higher electron density, allowing visualization of their localization at the oil–water interface. In the control emulsions (Fig. 10A), the protein particles were irregularly distributed and did not fully cover the droplet surfaces. Consequently, the exposed interfacial regions permit direct droplet contact and coalescence, ultimately compromising the long-term stability of the emulsion [59].

In contrast, the HU-treated samples (Fig. 10B and C) exhibited a more uniform and continuous interfacial coverage in both groups of HIPPEs. This improved interfacial adsorption could be attributed to the increased solubility and H_o_ content induced by the treatment. The resulting denser and more cohesive interfacial layers illustrate an important mechanism of Pickering emulsion stabilization, in which irreversibly adsorbed protein particles form robust steric barriers that minimize interfacial gaps and protect the droplets from coalescence, particularly under mechanical stress or during long-term storage [2].

Although both proteins underwent structural changes upon HU treatment, the higher solubility and smaller particle size observed in the FBPIs likely facilitated tighter interfacial packing and greater coverage density. This suggests that FBPI may contribute to a more effective stabilization of HIPPEs, particularly under conditions that demand improved interfacial integrity. These observations are also consistent with the secondary structural changes described in Section 3.1.8, where HU treatment was associated with an increase in α-helix content and a reduction in aggregation. Such conformational modifications may contribute to protein flexibility and promote more efficient rearrangement and anchoring at the oil–water interface, ultimately enhancing emulsion stability.

These microscopic observations were further supported by a 30-day storage study at 4 °C, the visual outcomes of which are shown in Fig. S1. As HIPPEs possess a densely packed droplet network and extremely high apparent viscosity, macroscopic phase separation remained minimal across all samples. This structural feature limits the sensitivity and interpretability of conventional stability indices such as creaming percentage or backscattering profiles. Consistent with this, no visible oil layer was observed, and only a thin cream layer appeared in FCON and PCON after 30 days of storage. Notable creaming in these controls indicated poor long-term stability of HIPPEs stabilized with untreated proteins, with Fig. 10A showing incomplete interfacial coverage and droplet coalescence. In contrast, the HU5 and HU10 treated HIPPEs maintained a homogeneous appearance without visible phase separation throughout the storage period, reflecting enhanced physical stability. These results suggest that moderate HU treatment (5–10 min) promoted the formation of a more cohesive interfacial layer, likely owing to improved solubility and Ho, which collectively increased the interfacial adsorption efficiency. Interestingly, HIPPEs stabilized with HU20 treated proteins exhibited slight creaming after 30 days, despite the application of the same physical modification. This observation aligns with the D_4,3_ measurements (Table 2), the HU20 treated samples exhibited larger droplet sizes than HU10 treated ones. Therefore, the reduced stability of HU20 may be attributed to an insufficient interfacial packing density and decreased surface area coverage by larger droplets, which can diminish steric hindrance and facilitate gravitational separation [60].

#### 3.2.3 Interfacial protein adsorption and packing behavior in HIPPEs

**Fig. 11:**
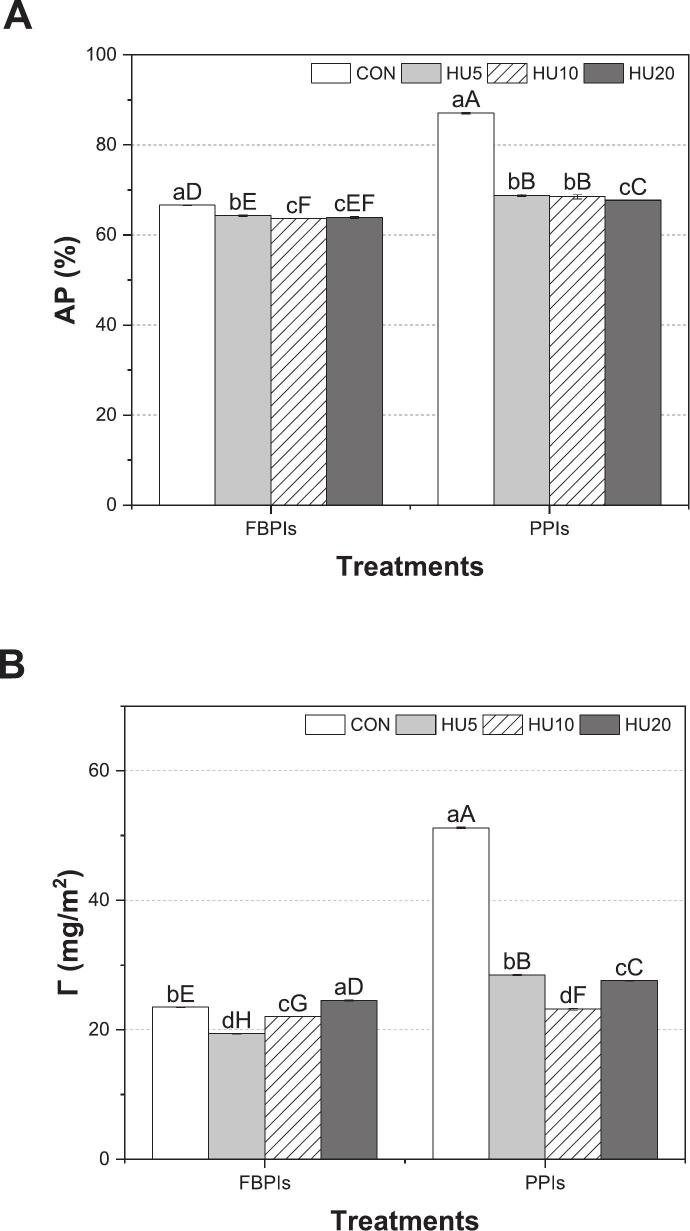
Adsorbed protein at interface (A) and interfacial protein concentration (B) of HIPPEs stabilized by HU-treated legume protein isolates. HIPPEs, high internal phase Pickering emulsion; FBPIs, faba bean protein isolates; PPIs, pea protein isolates; CON, untreated 4 % protein dispersions; HU, 4 % protein dispersions sequentially heated and ultrasonicated for 5, 10 and 20 min, respectively. ^a–d^ Data labeled with different lowercase letters indicate a significant difference among HIPPEs stabilized by different legume proteins (*p* < 0.05). ^A–^^H^ Data labeled with different uppercase letters indicate a significant difference across all samples (*p* < 0.05).

Pickering emulsions stabilized by protein particles benefit from irreversible adsorption at the oil–water interface, forming robust steric barriers that inhibit droplet coalescence [61]. To evaluate the interfacial behavior of protein particles in HIPPEs, both the percentage of adsorbed protein at the oil–water interface (AP, %) and interfacial protein concentration (*Γ*, mg/m^2^) were analyzed (Fig. 11). The AP represents the overall extent of protein accumulation at the interface and reflects the ability of particles to form steric barriers that contribute to emulsion stability via repulsive forces [62]. Meanwhile, *Γ* quantifies the mass of protein per unit interfacial area and is strongly associated with film thickness and density, higher *Γ* values typically correspond to enhanced resistance to coalescence due to the formation of more cohesive interfacial films [28].

As demonstrated by [23], [63], AP is closely associated with protein solubility, reflecting its influence on interfacial adsorption behavior. FCON and PCON, which exhibited the lowest solubility (Fig. 2A) among all treatments (*p* < 0.05), had the highest AP values of 66.63 % and 87.05 %, respectively (*p* < 0.05). This indicates that poor dispersibility in the aqueous phase may promote passive accumulation of aggregated proteins at the interface. Upon HU treatment, the AP decreased slightly in the FBPI group (63.7 %) and more substantially in the PPI group (67.7 %), coinciding with a progressive increase in solubility (*p* < 0.05). These results suggest that the increased solubility due to HU treatment may reduce the tendency of proteins to accumulate at the interface, possibly by promoting greater molecular dispersion in the continuous phase and weakening the driving force for adsorption.

While HU treatment decreased AP values, this does not necessarily indicate reduced adsorption capacity. Rather, it reflects a shift toward more efficient interfacial organization. In HIPPEs, stability is governed not by the total adsorbed amount but by the uniformity, continuity, and mechanical coherence of the adsorbed layer. Large aggregates in control samples may artificially raise AP and *Γ* values because they adhere in clusters, yet they create patchy, discontinuous coverage that leaves parts of the interface exposed and prone to coalescence. Such aggregation can also impose steric hindrance that limits effective adsorption at the oil–water interface, as reported for mixed protein systems [73]. However, HU treatment produced smaller and more mobile protein particles capable of adsorbing homogeneously across the oil–water interface, forming cohesive and elastic interfacial films that resist deformation. This was clearly supported by CLSM and TEM micrographs (Fig. 9, Fig. 10), which showed continuous and uniformly distributed protein layers after HU treatment despite lower AP and *Γ* values. Therefore, the apparent reduction in AP and *Γ* should be interpreted not as diminished adsorption capacity, but as a shift toward more efficient and structurally coherent interfacial organization.

**Fig. 12:**
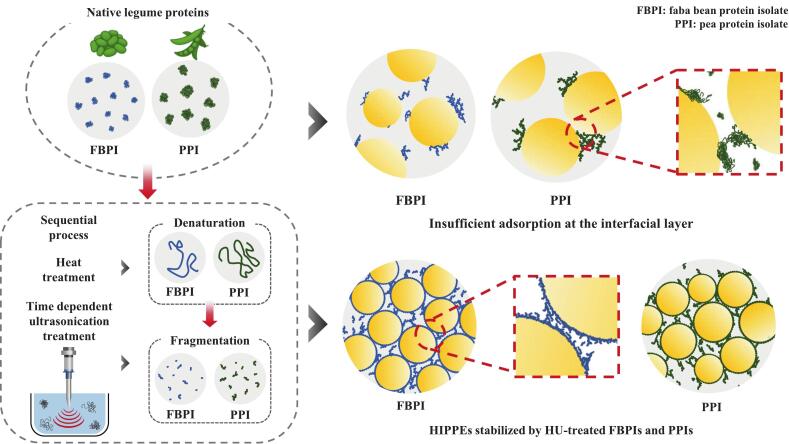
Schematic illustration of the proposed mechanism underlying HU-treated legume protein-stabilized HIPPEs. HIPPEs, high internal phase Pickering emulsion; FBPIs, faba bean protein isolates; PPIs, pea protein isolates; HU, 4 % protein dispersions sequentially heated and ultrasonicated for 5, 10 and 20 min, respectively.

In contrast to AP, *Γ* did not follow a linear behavior. For HIPPEs stabilized by FBPIs, *Γ* decreased to 19.40 mg/m^2^ after HU5, recovered to 22.03 mg/m^2^ at HU10, and exceeded the control at HU20 (24.54 mg/m^2^). For PPIs, the highest *Γ* value was observed in PCON (51.19 mg/m^2^), and dropped sharply to 23.18–28.48 mg/m^2^ after HU treatment, without full recovery. This contrasting behavior between AP and *Γ* suggests that improved dispersion and increased interfacial area, rather than total protein loading, promote tighter interfacial packing in HU-treated samples. This indicates that stability can be achieved through more efficient utilization of proteins, where smaller amounts of well dispersed particles are sufficient to form cohesive and uniform interfacial films. Olsmats et al. (2025) [74] have also reported that once the interface becomes saturated, additional protein adsorption no longer enhances stability, and the viscoelastic properties of the interfacial film become the dominant determinant of long-term resistance to coalescence. Such enhanced interfacial organization was reflected in finer and more uniform droplet size distributions and higher viscoelastic moduli of HU-treated HIPPEs (Table 2 and Fig. 12), confirming that stability arises from optimized interfacial packing and film elasticity rather than total protein load. This interpretation is further supported by the higher D_4,3_ values observed in HU-treated HIPPEs (Table 2), consistent with previous reports that interfacial area can lead to elevated *Γ* values [61], [64]. In contrast, the notably high *Γ* observed in PCON-stabilized HIPPEs may be related to the intrinsic nature of native PPI, which comprises oligomeric, structurally ordered globulins such as 7S and 11S. These proteins are known to form cohesive, shear-resistant interfacial films because of their compact tertiary structure and high molecular weight [65], resulting in dense, but possibly less dynamic, accumulation at the oil–water interface. A similar pattern was also observed in PHU5, PHU10, and PHU20, which exhibited slightly higher AP and *Γ* values than their FBPI counterparts. However, despite these elevated interfacial adsorption metrics, PPI-stabilized emulsions developed larger droplet sizes during storage and ultimately displayed inferior long-term stability. This discrepancy indicates that higher AP and *Γ* values in PPI-based systems do not necessarily translate into improved emulsion stability. Instead, the more flexible and adaptable interfacial layers formed by HU-treated FBPI sustained finer droplet distributions and greater structural integrity during storage, underscoring its superior robustness as a stabilizer in high internal phase Pickering emulsions.

These results indicate that HU treatment exerts its effect on interfacial behavior through a coupled mechanism involving colloidal stability, molecular conformation, and interfacial packing rather than through a single dominant parameter. The reduction in particle size and enhanced solubility increased protein diffusivity and dispersion uniformity, facilitating more homogeneous adsorption at the oil and water interface. Concurrently, the decrease in ζ-potential magnitude reduced electrostatic repulsion, enabling closer packing and cooperative rearrangement among adsorbed molecules. The observed partial unfolding and increased random coil content provided additional molecular flexibility, promoting the formation of cohesive and elastic interfacial films. Quantitative correlations between droplet size (D_4,3_), viscoelastic modulus (G′), and interfacial tension demonstrated that mechanical integrity and film continuity, rather than total protein load, are the primary determines of HIPPE stability. As illustrated in Fig. 12, HU treatment modulates the colloidal and structural attributes of legume proteins, such as solubility, particle size, and surface charge, which in turn influence how protein particles adsorb and organize at the oil–water interface. Enhanced dispersion promotes more uniform and compact interfacial coverage, compensating for the reduced AP and contributing to improved steric stabilization. Notably, the smaller droplet size and denser interfacial layer observed in HU-treated FBPI-stabilized HIPPEs underscore the stronger interfacial interactions of FBPI compared to PPI. These results highlight the importance of protein structural integrity and spatial distribution in forming cohesive interfacial films, offering mechanistic insights into how physical structuring can be leveraged to improve legume protein functionality in high-oil emulsion systems.

#### 3.2.4 Viscoelastic properties of HIPPEs

**Fig. 13:**
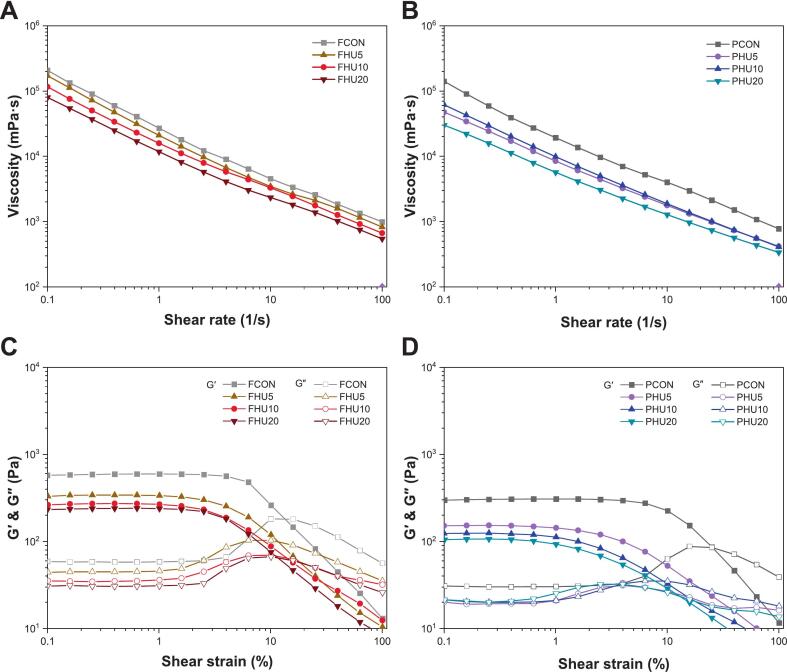
Viscosity (A, B) and storage modulus (G′), loss modulus (G″) (C, D) of HIPPEs stabilized by HU-treated legume protein isolates. HIPPEs, high internal phase Pickering emulsion; F, faba bean protein isolates; P, pea protein isolates; CON, untreated 4 % protein dispersions; HU, 4 % protein dispersions sequentially heated and ultrasonicated for 5, 10 and 20 min, respectively.

Understanding the flow behavior of HIPPEs is essential for evaluating their processability and textural performance in food applications [66]. As shown in Fig. 13A and B, all HIPPEs exhibited shear-thinning behavior, with apparent viscosity decreasing progressively as the shear rate increased from 0.1 to 100 s^−1^. This pseudoplastic response is a characteristic of emulsions and is attributed to the structural realignment and breakdown of droplets under shear [67]. Among the samples, emulsions stabilized by untreated proteins showed the highest viscosities, whereas HU-treated samples exhibited a clear reduction. Notably, the HU20-stabilized HIPPEs exhibited the lowest viscosities in both protein groups. This decrease may be explained by the smaller particle size of HU-treated proteins, which reduced steric hindrance and allowed for greater droplet mobility within the continuous phase [68]. Furthermore, the formation of thinner and less cohesive interfacial layers from more flexible protein particles may contribute to lower viscosity.

Fig. 13C and D show the storage modulus (G′) and loss modulus (G″) of HIPPEs obtained from amplitude sweep measurements. Within the linear viscoelastic region (LVR), all samples demonstrated solid-like behavior, as evidenced by G′ consistently exceeding G″. This implies that a stable internal network structure is formed by densely packed droplets and adsorbed protein layers [69], [70]. Beyond the LVR, both moduli decreased with increasing strain, which is characteristic of type III nonlinear behavior [71]. This strain-softening behavior suggests the disruption of droplet flocculation and the partial collapse of the interfacial film, weakening the gel-like structure under deformation [72].

In the FBPI group, G′ values decreased in the order of FCON > FHU5 > FHU10 > FHU20, and in the PPI group, PCON > PHU10 > PHU5 > PHU20. These results closely mirror the apparent viscosity data (Fig. 13A and B), in which the native protein-stabilized emulsions exhibited the highest viscosity. The higher G′ values in control groups may be due to larger protein particles forming more rigid droplet networks that limit mobility and enhance mechanical strength.

Notably, PHU10 HIPPEs exhibited the highest G′ among the modified PPI samples, which correspond to its smallest average droplet size (Table 2). This observation agrees with previous findings that smaller droplet sizes lead to a greater storage modulus owing to tighter droplet packing and stronger interfacial structures [66], [70]. A similar behavior was observed in the FBPI group, where FHU5 and FHU10, having smaller droplet sizes than FHU20, displayed higher G′ values, further reinforcing the relationship between reduced droplet size and improved viscoelastic performance.

## 4 Conclusion

This study demonstrated that sequential heat and ultrasound (HU) treatment effectively restructured the colloidal and interfacial properties of legume proteins, with ultrasonication duration identified as the decisive factor influencing particle size reduction, solubility enhancement, secondary structure rearrangement, and aggregation alleviation. HU-treated proteins improved the stabilization of high internal phase Pickering emulsions (HIPPEs) by enhancing colloidal dispersion and surface activity, leading to finer droplet formation, more uniform interfacial adsorption, and denser particulate films at the oil–water interface. Importantly, even though the adsorbed protein at interface (AP), and interfacial protein concentration (*Γ*) values decreased after treatment, the resulting films exhibited higher continuity and elasticity, indicating that stability in HIPPEs is governed more by film quality and droplet packing than by total interfacial protein load. Furthermore, HIPPEs stabilized by HU-treated proteins exhibited lower viscosities and improved viscoelastic properties, indicating the development of a flexible yet stable network structure. Comparing the two proteins, faba bean protein isolates (FBPIs) displayed superior adaptability to HU treatment compared with pea protein isolates (PPIs), as reflected in their higher solubility and smaller particle size. Although PPIs exhibited higher *Γ*, their emulsions showed reduced long-term stability during storage, highlighting the superior robustness of FBPI in sustaining interfacial structures over time. Collectively, these findings underscore the effectiveness of HU modification lies not only in improving colloidal properties, but also in shifting the stabilization mechanism from high protein loading to efficient interfacial film organization. This mechanistic base understanding establishes FBPI as a particularly promising clean-label emulsifier for high-oil systems, while also providing a mechanistic framework that can guide the selection and processing of other plant proteins. Future studies should further examine long-term storage stability, oxidative resistance, and encapsulation efficiency in complex food matrices.

## CRediT authorship contribution statement

**Hyo Gyeong Lee:** Writing – original draft, Visualization, Software, Methodology, Investigation, Formal analysis, Data curation. **Jiseon Lee:** Writing – review & editing. **Yeon-Ji Jo:** Formal analysis, Conceptualization. **Mi-Jung Choi:** Writing – review & editing, Supervision, Project administration, Conceptualization.

## Declaration of competing interest

The authors declare that they have no known competing financial interests or personal relationships that could have appeared to influence the work reported in this paper.
