# Supplementary material for: Ultrasonication time dependent structuring of heat-treated legume proteins: interfacial adsorption and stabilization of faba bean and pea protein isolates in high internal phase Pickering emulsions
Source: Ultrason Sonochem. 2025 Oct 30;123:107659. doi: 10.1016/j.ultsonch.2025.107659 (PMC12637073; doi:10.1016/j.ultsonch.2025.107659)
Supplement: Supplementary Data 1 [file mmc1.docx]

**Supplementary material**


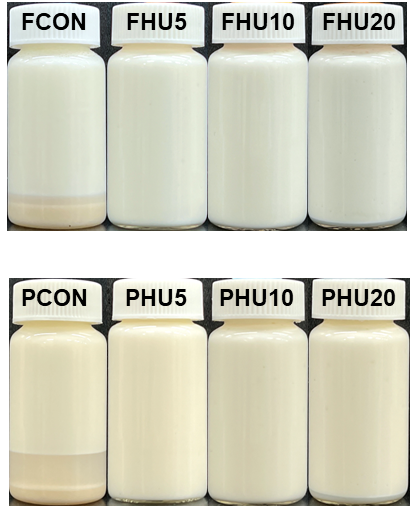


**Figure S1.** Visual appearance of HIPPEs stabilized by HU-treated legume protein isolates during storage at 4 °C for 30 days. HIPPEs, high internal phase Pickering emulsion; F, faba bean protein isolate; P, pea protein isolate; CON, untreated 4% protein dispersions; HU, 4% protein dispersions sequentially heated and ultrasonicated for 5, 10 and 20 min, respectively.
